# Vitrification of Molten Chloride Salts

**DOI:** 10.1021/acs.jpcc.6c03825

**Published:** 2026-09-14

**Authors:** Ellie M. Kim, Phillip W. Halstenberg, Xin Wang, Dmitry Maltsev, Shannon M. Mahurin, Aaron G. Penders, Kaustubh Bawane, James F. Wishart, Sheng Dai

**Affiliations:** † Department of Chemistry, 6146 The University of Tennessee, Knoxville, Tennessee 37996, United States; ‡ Chemical Sciences Division, Oak Ridge National Laboratory, Oak Ridge, Tennessee 37830, United States; § Nuclear Materials Performance Division, Idaho National Laboratory, Idaho Falls, Idaho 83415, United States; ∥ Chemistry Division, Brookhaven National Laboratory, Upton, New York 11973, United States

## Abstract

Vitrification of molten chloride salts offers a novel approach for preserving their liquid-phase structure as amorphous solids, enabling analysis of liquid structure at room temperature. However, vitrifying molten salts composed of simple salts (e.g., NaCl, KCl) has remained a significant technical challenge due to their strong tendency to crystallize. In this study, a nebulizer was used to successfully vitrify a KCl–MgCl_2_ mixture for the first time. Powder X-ray diffraction (pXRD) analysis confirmed a substantial reduction in crystallinity, indicating enhanced amorphization of the quenched salts. Four-dimensional scanning transmission electron microscopy (4D-STEM) revealed retention of the amorphous state. Raman spectroscopy confirmed the persistence of tetrahedral [MgCl_4_]^2–^ motifs in the room-temperature solids, demonstrating preservation of molten-state structural features in the vitrified phase. This work represents a critical advancement in molten salt vitrification, providing a new pathway for structural studies and informing future applications in molten salt reactors and high-temperature chemical processes.

## Graphical abstract

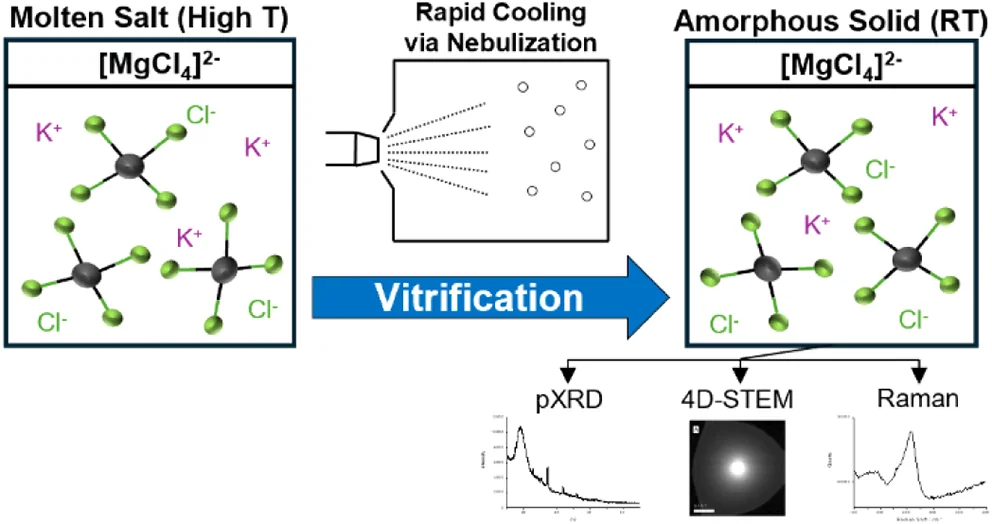

## 1 Introduction

Glass is an amorphous solid which is a unique material that can be viewed from a chemical structure perspective as a liquid with nearly infinite viscosity.^1^ In contrast, crystalline materials consist of well-defined unit cells that do not reflect the liquid state.^2^ When a material is vitrified, it undergoes a phase transition from liquid to solid before atoms can arrange into a crystalline structure. This process typically involves supercooling to suppress atomic rearrangement and preserve the disordered structure of the liquid. Vitrification has been widely utilized in scientific applications such as cryopreservation.^3^ In 1981, Dubochet successfully vitrified pure water for the first time via quenching in liquid propane, paving the way for advances in molecular biology and drug discovery.^4^ This breakthrough helped establish the field of cryobiology, whereby vitrification enables microscopic analysis of cells and tissues without inducing damage from water crystallization, a technique that underpins the development of cryogenic electron microscopy.^5^ The development of cryo-EM ultimately contributed to the 2017 Nobel Prize in Chemistry awarded to Jacques Dubochet, Joachim Frank, and Richard Henderson.^6^

The unique properties of glass provide a pathway for analyzing and characterizing solid materials while retaining aspects of their liquid-phase structure, enabling the study of high-melting point materials at room temperature. Molten salts, composed of ionic compounds, typically exhibit melting points above 100 °C. The required high temperatures pose significant challenges for experimental characterization of the liquid state.^7^ In addition to the high temperature, additional challenges arise from their corrosive and hygroscopic nature. These result in complex handling requirements and increased safety hazards that limit available spectroscopic techniques and suitable containment vessels for other analytical approaches.^8^ Such challenges are compounded by moisture absorption and metal oxide formation during high-temperature experiments. Performing analyses of molten salts at room temperature would alleviate many of these experimental difficulties. However, room-temperature analysis of molten-state characteristics has not been feasible, due to complete crystallization upon solidification.^9–12^

The process of extreme supercooling very rapidly removes the energy needed to overcome the activation barrier to nucleation, thereby inhibiting the formation of crystalline structures and enabling glass formation in metal chloride mixtures.^13^ This phenomenon aligns with nucleation theory and is further described by the Vogel–Tammann–Fulcher equation shown in eq 1, which relates viscosity and temperature in the context of nucleation,

1 $$\log\eta =A+\frac{B}{T-T_{0}}$$

where η is the material’s viscosity, *A* is the pre-exponential factor describing baseline viscosity, *B* is a material-dependent constant related to the activation energy required for molecular motion in a viscous liquid, *T* is the absolute temperature of the system, and *T*_0_ is the Vogel’s temperature, at which viscosity is theoretically infinite.^14–16^ Previous studies that successfully vitrified molten salts have focused on ZnCl_2_ or ZnCl_2_-based systems, as ZnCl_2_ is a well-known glass former due to its high viscosity.^17–19^ Efforts to vitrify molten salts not containing ZnCl_2_ have employed liquid argon (boiling point −187 °C),^20^ which, due to the Leidenfrost effect slowing solidification, only slightly reduces crystallization.^10^ This effect creates an insulating vapor layer that slows cooling and inhibits the formation of glass from molten salt.^21^ In this study, a 68:32 mol % KCl-MgCl_2_ eutectic was vitrified by rapidly cooling fine droplets generated using a nebulizer into a liquid nitrogen (LN_2_)-cooled fused silica tube.

A key distinction of this study is that vitrifying molten salts involves a significantly larger temperature gradient than vitrifying water using liquid propane (boiling point −42 °C),^22^ which does not involve the Leidenfrost effect. In this work, the molten salt temperature was maintained at 550 °C, while the collection tube was held at LN_2_ temperature (−196 °C). This extreme temperature gradient, combined with the high-velocity argon gas flow generated by the nebulizer through the Venturi effect, enabled rapid solidification that preserved the disordered structure of the molten salt. This novel approach to amorphization can facilitate future studies of the properties of liquid-state salts at room temperature, opening new avenues for material characterization in high-temperature chemistry.

## 2 Experimental Section

### 2.1 Materials

Anhydrous 99.999% trace metals grade KCl was purchased from Sigma-Aldrich and used after drying under vacuum at 500 °C for 1 h. MgCl_2_ (anhydrous, 99.9% purity) was purchased from Sigma-Aldrich and purified as described previously.^23^

### 2.2 Apparatus

The Collison nebulizer was purchased from CH Technologies USA Inc. The salt mixture was loaded into a fused silica tube inside a stainless-steel standard Collison jar inside an inert atmosphere glovebox (MBraun LABstar Pro, AirGas UHP nitrogen <0.5 ppm of O_2_ and H_2_O). A titanium nozzle attached to the lid’s aperture was assembled with the jar. The system was removed from the glovebox after sealing the gas inlet and the aerosol outlet with Parafilm. The nebulizer was then connected to an argon gas line, and the entire system with the salt was continuously maintained under an argon atmosphere throughout the nebulization process to minimize exposure to ambient moisture. The collection tube was connected to the aerosol outlet using a hollow silicone stopper. The collection tube was placed inside a container filled with LN_2_, which was continuously replenished throughout the experiment. Once the salt was fully molten inside the nebulizer, the argon gas pressure was set to 40 psi, and the nozzle was immersed into the molten salt. After collection was completed, the tube was quickly transferred back into the drybox. A schematic diagram of the experimental apparatus is provided in Figure S1.

### 2.3 Analysis

Powder X-ray diffraction (pXRD) measurements were performed using a PANalytical Empyrean X-ray diffractometer with Cu Kα radiation (λ = 1.5406 Å). Inside an argon-filled glovebox, samples were transferred to a domed sample holder purchased from Malvern Panalytical, which maintained an inert atmosphere during XRD measurements. No MgO was detected in the XRD patterns of samples prepared using this procedure, indicating that significant moisture uptake during the experiment was not evident. Raman spectra were collected using a Renishaw inVia confocal Raman microscope with 532 nm excitation. Inside the glovebox, samples were transferred to clear vials, sealed with electrical tape, and stored in a desiccant-containing jar until measurement. These precautions minimized moisture exposure during sample preparation and subsequent characterization. Additional details of the Raman measurements are provided in the SI. Four-dimensional scanning transmission electron microscopy (4D-STEM) analysis was performed using an FEI Titan ChemiSTEM FEG-STEM. The vitrified KCl-MgCl_2_ powder was stored under the same conditions used for Raman sample preparation until it was loaded onto a copper grid for analysis.

## 3 Results and Discussion

### 3.1 Powder X-ray Diffraction (pXRD) Analysis

The crystalline phases present in the KCl-MgCl_2_ eutectic before and after vitrification were analyzed using pXRD. As shown in Figure 1, the pXRD diffractogram prior to vitrification indicates a crystalline phase corresponding to K_2_MgCl_4_, along with a background peak at 2θ=43°.^25^ Following vitrification, the KCl-MgCl_2_ system exhibited diffuse scattering with broadened peaks and significantly diminished peak intensity.

**Figure 1:**
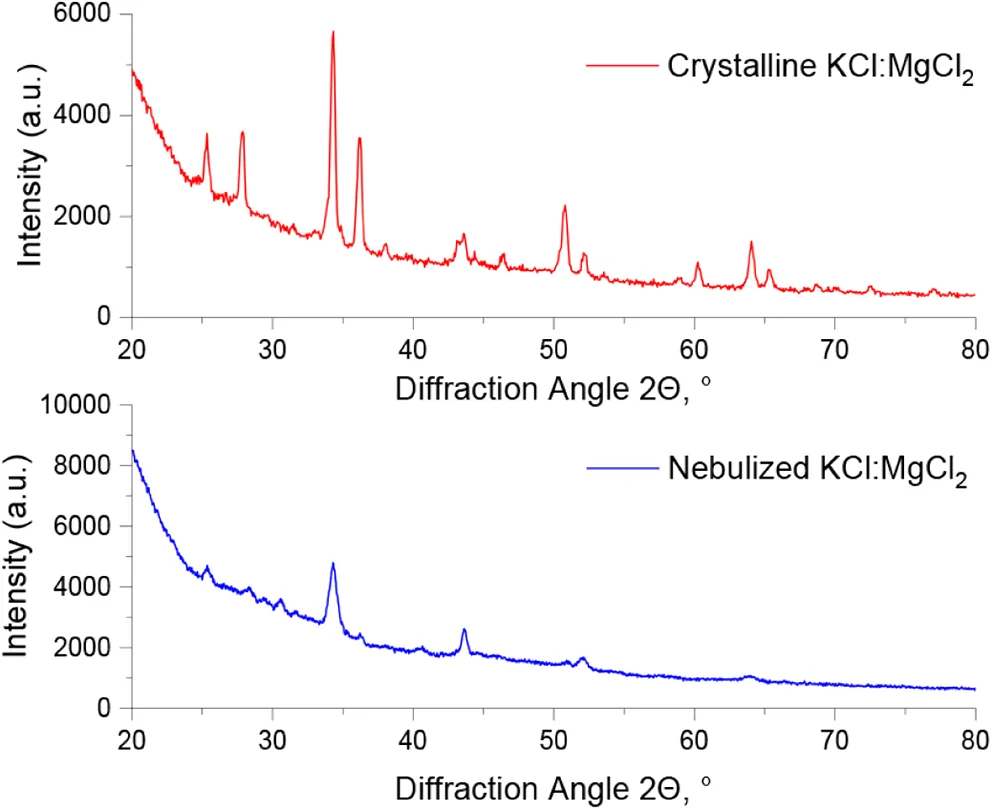
Powder X-ray diffraction (pXRD) diffractograms of KCl-MgCl_2_ eutectic cooled from molten state slowly (red) and after vitrification (blue).

The measured diffractograms indicate that vitrification effectively suppressed crystallization, resulting in an increased amorphous character and a reduction in detectable crystalline phases.

### 3.2 Raman Spectroscopic Analysis

Raman spectroscopy was used to evaluate the structural changes in the KCl-MgCl_2_ eutectic between the crystalline and amorphous states by identifying vibrational modes associated with distinct chemical species. There is a significant structural difference between solid and liquid KCl-MgCl_2_ systems. The high-temperature liquid phase contains some tetrahedral [MgCl_4_]^2–^ motifs, whereas the solid phase adopts the cubic perovskite-type K_2_MgCl_4_ structure where all the Mg^2+^ ions are octahedrally coordinated, with a sharp Raman peak at ∼200 cm^–1^ as shown on the left side of Figure 2.^24–28^ The Raman spectrum of vitrified KCl-MgCl_2_, shown in Figure 2 right, exhibits a prominent peak at 253 ± 3 cm^–1^, corresponding to the symmetric stretching A_1_ mode of the tetrahedral [MgCl_4_]^2–^ motif.^25,26^ The broad, diffuse band around 200 cm^–1^ is attributed to solid K_2_MgCl_4_, which is consistent with the residual crystalline phase observed in the diffractogram after vitrification shown in Figure 1.

**Figure 2:**
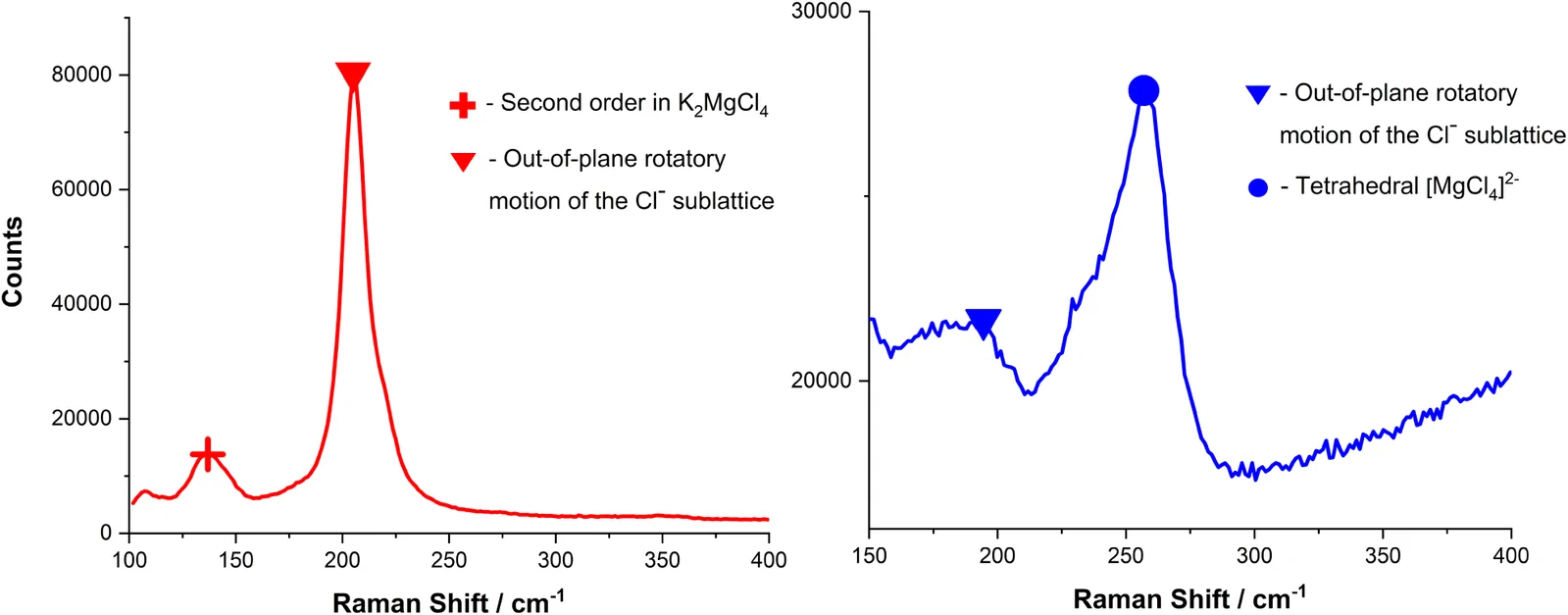
Raman spectra of KCl-MgCl_2_ eutectic cooled from molten state slowly (red) and after vitrification (blue).

The existence of tetrahedral [MgCl_4_]^2–^ motifs represents a distinctive signature indicating the preservation of the local structure of the liquid state upon vitrification through the rapid quenching process employed in this work. Similar structural motifs have been reported in other chloride systems, including CdCl_2_–KCl, CsCl-MgCl_2_, RbCl-MgCl_2_, MgCl_2_–NaCl, and MgCl_2_–LiCl.^25,29^ By contrast, slow cooling typically results in the crystalline phase, showing the 200 cm^–1^ band rather than preserving tetrahedral Mg^2+^ coordination.^25^

### 3.3 4D Scanning and Transmission Electron Microscopy (4D-STEM) Analysis

To further confirm the amorphous nature of the vitrified salts, 4D-STEM was employed to obtain a comprehensive characterization of the particles. As shown in Figure 3, virtual selected area electron diffraction apertures were placed at six different locations (labeled A–F), of which the corresponding diffraction patterns exhibit diffuse rings across multiple particles without observable Bragg diffractions, confirming the absence of long-range crystalline order. Elemental mapping using energy-dispersive X-ray spectroscopy (EDS), shown in Figure 4, confirms that the particles consist of Mg, K, and Cl, consistent with the composition of the KCl-MgCl_2_ system.

**Figure 3:**
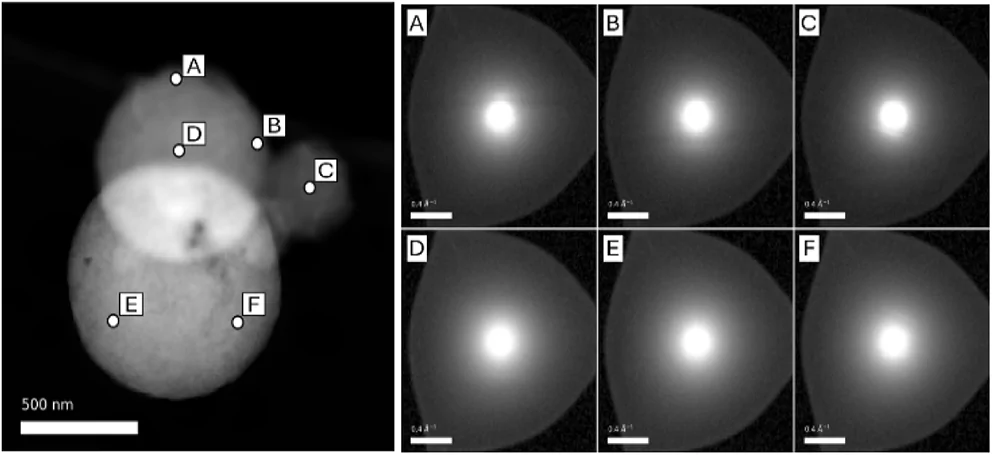
4D-STEM electron diffraction of vitrified 68:32 mol % KCl-MgCl_2_ at various spots labeled A–F.

**Figure 4:**
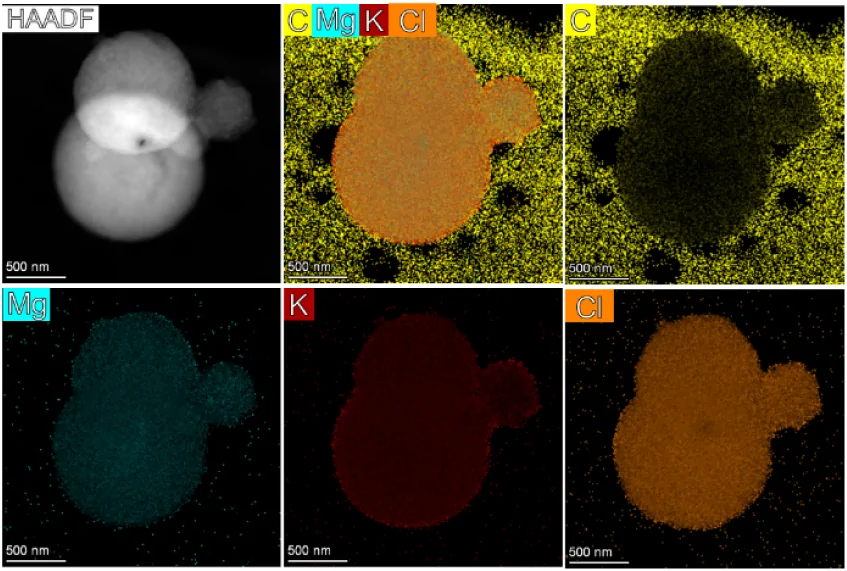
Super-X EDS elemental mapping of vitrified 68:32 mol % KCl-MgCl_2_.

Although the present study focuses on the KCl-MgCl_2_ binary system, the vitrification of a KCl-containing molten salt demonstrates the potential broader applicability of the nebulization approach to simple chloride salts. In particular, we anticipate that the method could be extended to other simple unary monovalent salts, such as NaCl and KCl, with appropriate modifications to the experimental setup to accommodate their higher melting points. The applicability of the approach to these systems remains to be established experimentally and represents an important direction in the ongoing work.

These results highlight the effectiveness of the nebulizer-based approach for vitrifying high-temperature molten salts, overcoming limitations of prior methods that relied on cryogenic quenching media. This advancement enables room-temperature structural characterization of molten salts, providing critical insights for applications in molten salt reactors and other high-temperature systems. Future work will focus on improving vitrification control, evaluating scalability for industrial applications, and extending the approach to a broader range of molten salt systems. Additional studies will also examine the thermal and chemical stability of vitrified salts as surrogates for probing the liquid-state structural features.

## 4 Conclusions

This study successfully demonstrates the vitrification of molten chloride salts using a nebulizer, providing a reliable method for preserving the liquid-phase structure of these salts in an amorphous solid state. An analysis with pXRD confirmed reduced crystallinity in the vitrified KCl-MgCl_2_ eutectic, as evidenced by the significant reduction in crystalline peak intensity following vitrification. Raman spectroscopy provided insight into structural transformations, revealing the existence of tetrahedral [MgCl_4_]^2–^ motifs in vitrified KCl-MgCl_2_, along with distinct vibrational modes associated with the amorphous state. Furthermore, electron diffraction analysis using 4D-STEM demonstrated that vitrified KCl-MgCl_2_ retained its amorphous structure across multiple particles, as indicated by diffuse rings and the absence of Bragg diffraction.

## Supplementary Material



## Data Availability

The data for all figures contained in this publication and Supporting information will be made accessible on the Zenodo repository under DOI: TBD.

## Notes

A schematic diagram of the experimental apparatus and XRD diffractograms showing the polycarbonate dome effect (PDF)
