# Supplementary material for: Vitrification of Molten Chloride Salts
Source: J Phys Chem C Nanomater Interfaces. 2026 Sep 14;130(41):14307–11. doi: 10.1021/acs.jpcc.6c03825 (PMC13679037; doi:10.1021/acs.jpcc.6c03825)
Supplement: Supplementary file 1 [file jp6c03825_si_001.pdf]

Vitrification of Molten Chloride Salts

Ellie M. Kim[a], Phillip W. Halstenberg[a,b], Xin Wang[b], Dmitry Maltsev[b], Shannon Mahurin[b], Aaron Penders[c], Kaustubh Bawane[c], James Wishart[d], Sheng Dai[a,b*]

[a] Department of Chemistry, The University of Tennessee, Knoxville, 37996, TN, United States

[b] Chemical Sciences Division, Oak Ridge National Laboratory, Oak Ridge, 37830, TN, United States

[c] Characterization and Advanced Post Irradiation Examination Division, Idaho National Laboratory, Idaho Falls, 83415, ID, United States

[d] Chemistry Division, Brookhaven National Laboratory, Upton, 11973, NY, United States

Molten salt, metal chloride, vitrification, quenching, glass, amorphous

1. Raman spectroscopy measurements

Raman spectra were acquired using a Renishaw InVia Raman spectrometer coupled to a Leica microscope. A 5× objective was used to focus 532 nm light from a solid-state laser onto the sample. Raman-scattered light was collected through the same objective and focused through a 2400 l/mm holographic grating onto a Peltier-cooled CCD camera. The sample was loaded into a borosilicate vial for the measurement, and the excitation laser was focused through the wall of the vial onto the sample.

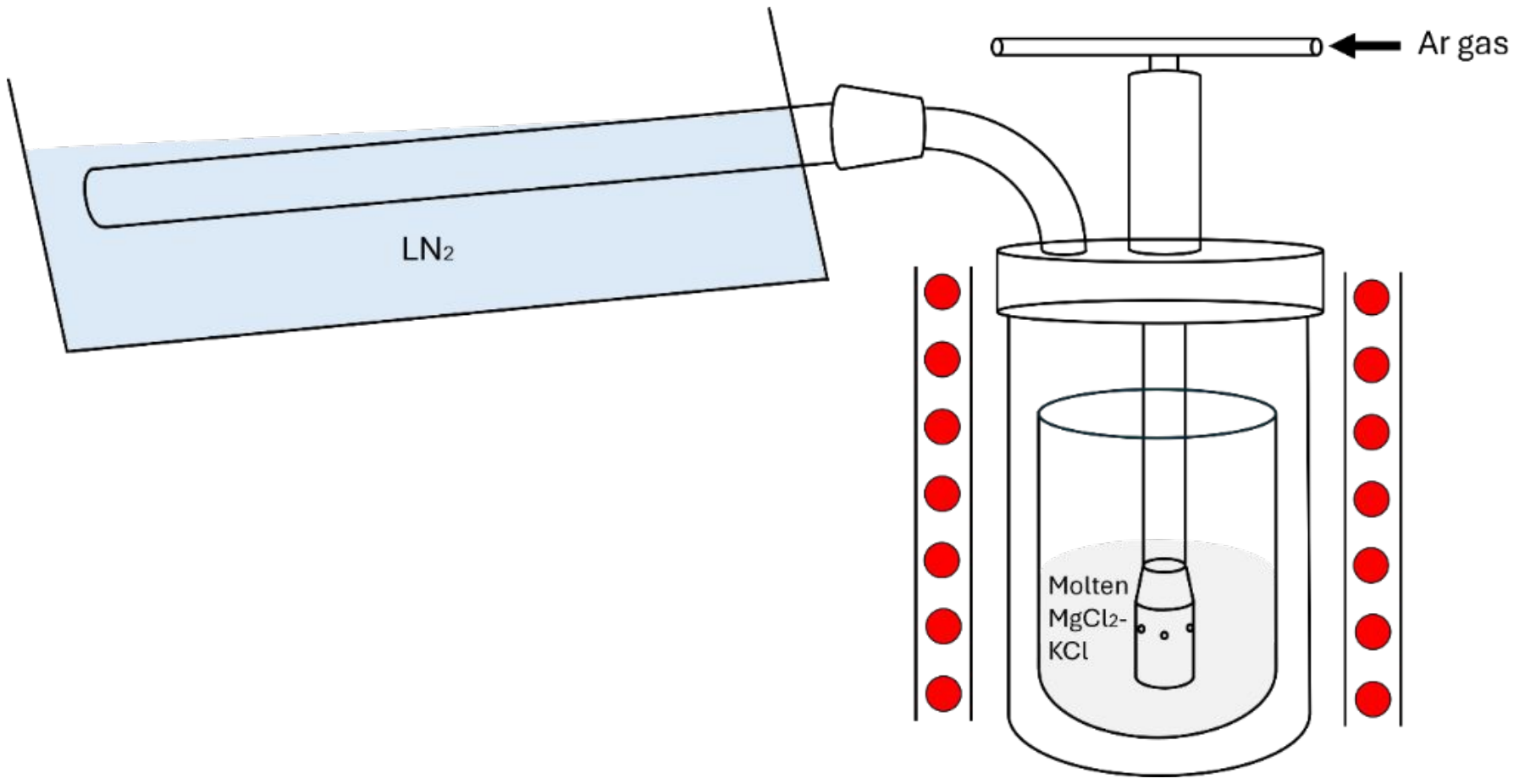

Figure S1. Schematic diagram of the experimental apparatus of molten salt vitrification.

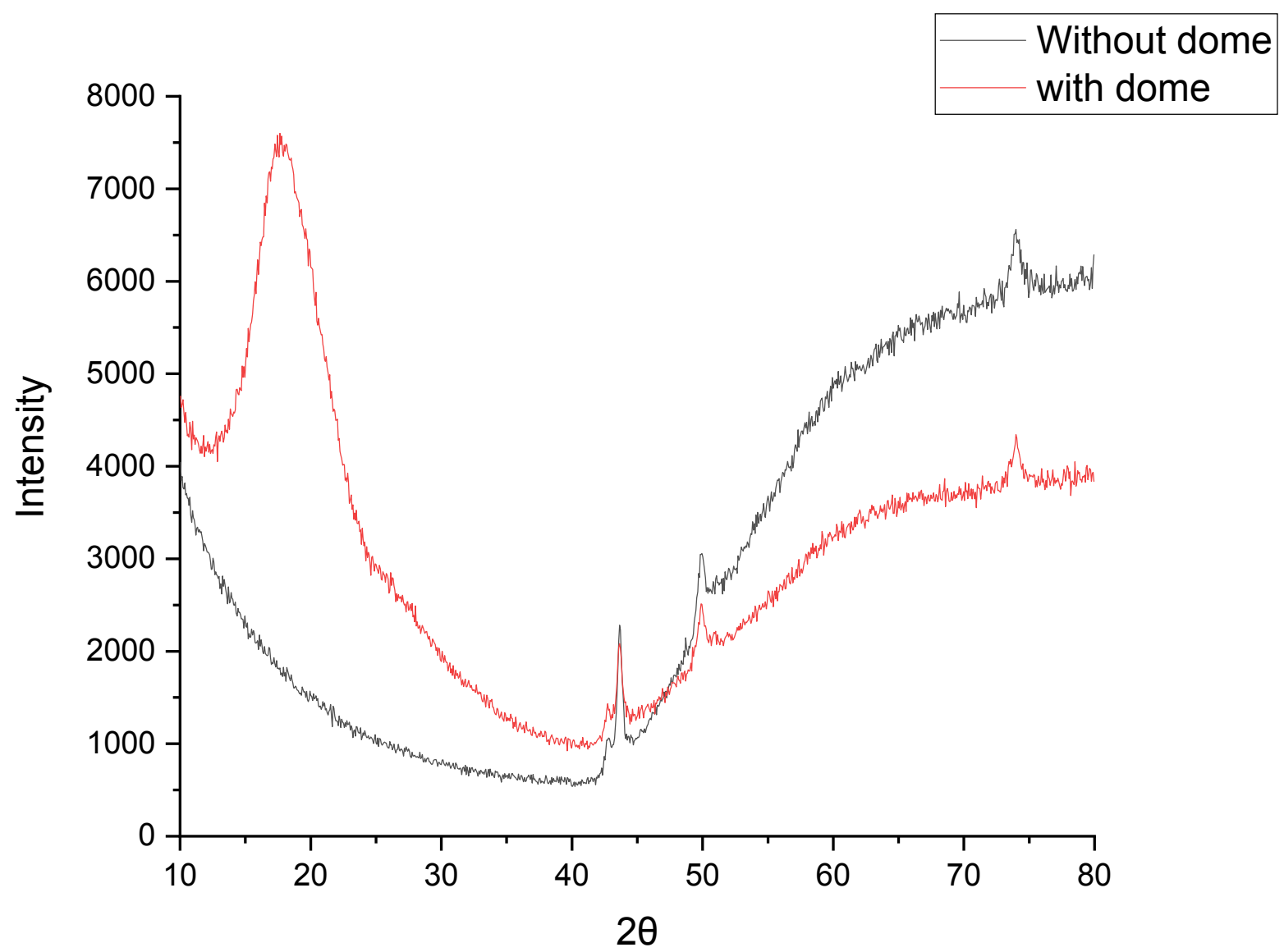

Figure S2. X-ray diffraction (XRD) spectra of a blank sample stage with the polycarbonate dome (red) and without the dome (black).
